# Evaluating the Effects of Carriere Motion Appliance and Twin Block Appliances in Class II Correction—A Retrospective Study

**DOI:** 10.3390/dj12050119

**Published:** 2024-04-23

**Authors:** Gilad Har Zion, Eyal Katzhendler, Amal Bader Farraj, Miryam Rabin, Shmuel Einy

**Affiliations:** 1 Private Practice of Orthodontics, Alfasi 19 St., Jerusalem 9230209, Israel; gilad9@012.net.il; 2 Department of Orthodontics, Faculty of Dental Medicine, Hebrew University-Hadassah School of Dental Medicine, Jerusalem 91120, Israel; askdreyal@gmail.com; 3 Galilee College of Dental Sciences, Department of Orthodontics, Galilee Medical Center, Nahariya 2210001, Israel; dr.amalbader@gmail.com (A.B.F.); dr.miryamrabin@gmail.com (M.R.); 4 The Azrieli Faculty of Medicine, Bar-Ilan University, Safed 5290002, Israel

**Keywords:** Class II malocclusion, Class II correction, Carriere Motion Appliance, twin block, incisor proclination

## Abstract

This retrospective study compared Class II orthodontic non-extraction treatment using Carriere Motion Appliance (CMA) and Twin Block (TB) appliances. Methods: The treatment of 38 patients was assessed. Pre- and post-treatment cephalometric radiographs were analyzed to evaluate skeletal, dental, and soft tissue treatment outcomes and efficacy. Results: Both appliances effectively corrected the Class II molar relationship. When measured at the distal aspect of the first molar, TB achieved 4.22 mm, while CMA had a 2.55 mm correction. When measured in the mesial aspect, the CMA achieved a 3.9 mm correction. The changes in SNB and ANB were statistically significant only in the TB group. The CMA appliance demonstrated statistically significantly less protrusion of the mandibular incisors and less upper incisor retrusion without vertical changes compared to the TB appliance. The TB demonstrated statistically significant lower lip protrusion compared to the CMA. Conclusion: The CMA corrects Class II malocclusions only by exerting a dentoalveolar influence and does not demonstrate the added effects associated with TB, such as elongation of lower facial height (LFH) and less loss of lower anchorage. Nonetheless, the correction in the TB group comprised both dentoalveolar and skeletal components. The CMA promotes a multidirectional upper and lower molar movement, and despite our 2D cephalometric analysis, we were able to estimate the extent of upper molar derotation.

## 1. Introduction

Class II malocclusion is a frequently encountered orthodontic challenge. It is characterized by the mesial position of the upper molars relative to the lower molars, accompanied by the protrusion of the upper incisors [1,2]. Various treatment modalities are available for Class II malocclusion correction, depending on the underlying cause and whether it involves a skeletal or dentoalveolar discrepancy [3,4]. Skeletal correction can be accomplished by using fixed appliances such as Forsus [5] or Herbst [6], removable functional appliances such as Twin Block (TB) [7], or the mandibular advancement appliance of Invisalign [8]. The TB appliance [7,9] induces significant skeletal changes, including mandibular advancement and posterior repositioning of the maxilla in the sagittal plane and an enlargement of the lower facial height in the vertical plane. Dentally, it corrects Class II malocclusion via the mesialization of the lower dentition and distalization of the upper dentition [7]. Nevertheless, a significant loss of anchorage was observed in patients treated with TB, including the forward movement of the incisors [10]. In addition, the use of the TB appliance may cause changes in the soft tissue, such as an improvement in profile convexity [6,7].

The correction of Class II malocclusion can also be achieved by upper molar distalization through various methods, including the Pendulum [11], Carriere Motion 3D™ appliance [12], and mini screws [13,14]. The Carriere Motion 3D™ appliance (CMA; Henry Schein Orthodontics, Carlsbad, CA, USA) was introduced in 2004. It is a partially fixed appliance that can effectively correct Class II malocclusions [12]. It is an intraoral appliance used as an alternative to orthodontic headgear for the distalization of maxillary molars [10]. It is compact, causes minimal discomfort, and is designed mainly for bodily movement rather than the tipping of the maxillary molars [15,16]. Along with its effective distalization of the maxillary molars, it might produce undesired effects, such as proclination and the protrusion of the mandibular incisors, molar tipping, and opening of the bite [16,17]. The ball-and-socket design of the CMA located in front of the molar pad, which is bonded to the maxillary first molars, facilitates distopalatal rotation around their palatal roots, resulting in the improvement of the sagittal relationship of the molars [12]. The use of Class II intermaxillary elastics can generate an anchorage loss manifested as the mesial movement of the lower dentition and protrusion of the lower incisors [18]. An Essix appliance, which holds all the mandibular teeth together, can mitigate this adverse effect [9]. A limited number of studies investigated the treatment effects of CMA and concentrated primarily on dental changes [19,20]. Others have examined dentoalveolar and skeletal alterations, with limited association with soft tissue [17,21,22]. Moreover, mandibular position changes and loss of anchorage induced by CMA were studied only in comparison to fixed functional appliances [18], while no study specifically compared the impact of CMA on removable functional appliances like TB. Therefore, the aims of this study are as follows:

1. To assess the effect of CMA treatment in Class II malocclusion cases on skeletal, dental, and soft tissue variables.
2. To compare the treatment effects of CMA and TB on Class II malocclusion.

Our null hypothesis is that the treatment effect of the CMA is mainly dentoalveolar.

## 2. Materials and Methods

This retrospective study compared 22 consecutive adolescent Class II patients treated using CMA to 16 consecutive adolescent Class II patients who were treated with TB, including 38 subjects altogether. A power analysis was conducted using G*Power software (version 3.1.9.6) based on a two-tailed *t*-test with a significance level of α = 0.5 and two independent group mean differences [22,23], aiming for a power of 0.8, which indicated that a total sample size of at least 34 subjects was sufficient.

All the patients were treated by two orthodontists. The study methodology entailed a comparative analysis of pre- and post-treatment cephalometric radiographs, which were taken at the beginning and at the end of the treatment phase with a CMA or a TB when a bilateral Class I molar occlusal relationship was achieved. The patients were collected retrospectively based on the following inclusion criteria: (1) late mixed dentition or permanent dentition at the beginning of treatment, (2) a bilateral angle Class II molar relationship with an end-to-end or larger molar relationship, (3) a bilateral Class II canine or premolar relationship with at least half a unit, and (4) all patients successfully completing the treatment and post-treatment with molar and canine Cl I relationships achieved. Patients with syndromes, skeletal deformities, and the unilateral use of CMAs were excluded. The study was approved by the ethics board committee (0693-19-RMB, 0346-19-RMB).

In the CMA group, the appliance was fitted according to the manufacturer’s instructions. The mandible buccal tubes with elastic hooks were bonded to the first molars. A clear retainer fabricated of 1 mm thick Essix A+ (Sirona Dentsply, Charlotte, NC, USA) plastic was used in the mandibular dentition for anchorage. The elastic wear comprised Force 1 elastics (1/4 inch 6 oz) for one month, followed by Force 2 elastics (3/16 inch 8 oz; Henry Schein Orthodontics Carlsbad, CA, USA), which were worn until a Class I occlusal relationship was achieved. The patients were instructed to wear the elastics all the time except during meals and tooth brushing and to change them after every meal.

**Figure 1:**
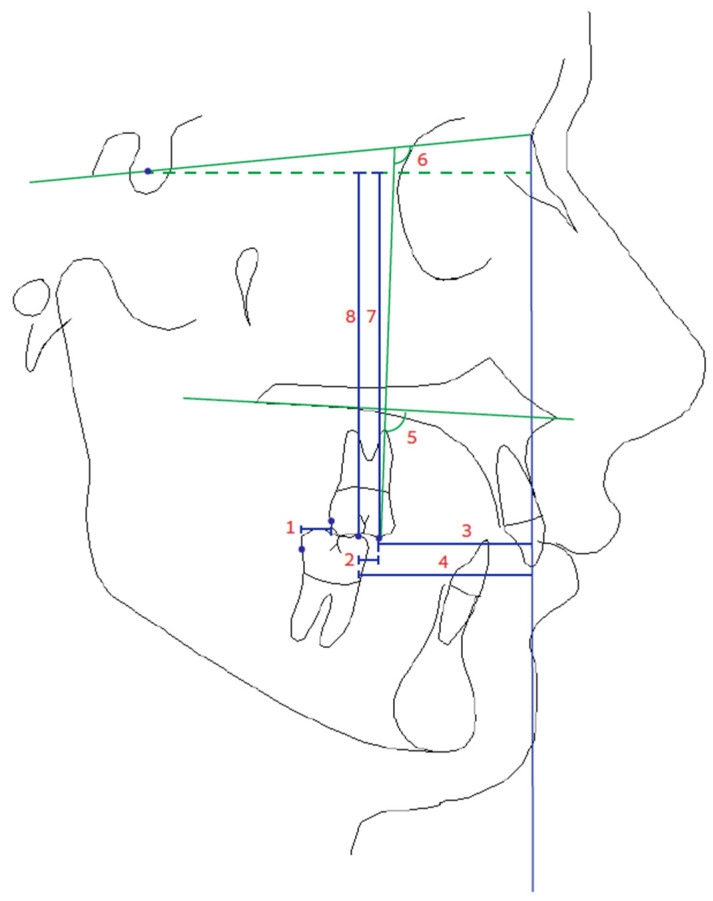
Cephalometric measurements for evaluating molars. 1. DAMR mm, 2. U6-L6 mm, 3. NP-U6 mm, 4. NP-L6 mm, 5. Palatal Pl—U6 °, 6. SN—U6 °, 7. S—U6 mm, and 8. S—L6 mm.

In the TB group, Adams clasps were constructed to anchor the appliance to the first permanent molars in both jaws. A short passive labial bow was incorporated into the lower arch between the canines, while torque springs were used for the upper incisors. The initial wax bite registration positioned the lower jaw forward to an edge-to-edge incisor relationship. In both groups, a second cephalometric radiograph was taken immediately at the end of the CMA or TB phase. All images were traced by a single investigator (E.K.). Each tracing was digitized using OrthoData Cephalometric Analysis Software version 5.0 (Ramat Denya, Jerusalem, Israel). Table 1 provides comprehensive definitions for the evaluated measurements. Seven additional parameters were analyzed to assess the impact of CMA on the molars for a thorough evaluation of the CMA effect (U6-L6, NP-U6, NP-L6, Palatal Pl—U6°, SN—U6°, S—U6, S—L6); see Figure 1. Statistical analysis was performed using PAST software v.4.09; Natural History Museum, University of Oslo, Norway (Hammer et al. 2001) [24]. The significance level was set at *p* < 0.05. General summary information for each measurement was obtained via descriptive statistics. The normal distribution of the samples was assessed using the Shapiro–Wilk test. For samples with a normal distribution, Student’s *t*-test was performed. When the distribution of the sample was not normal, a Mann–Whitney test with Bonferroni correction was carried out. For the comparison of treatment changes within the groups, a paired *t*-test was used for samples with normal distribution, and a Wilcoxon signed-rank test was used for samples without normal distribution.

## 3. Results

The average age of patients at the beginning of treatment with CMA was 14.15 ± 1.2 years, spanning a range of 11.03 to 15.09 years, with a median of 14.18 years. The average age of patients treated with TB was 12.15 ± 0.05 years, varying between 11.0 and 15.02 years, with a median age of 12.02 years. The average age between the two groups was significantly different (*p* < 0.01). The average treatment duration for CMA was 6.68 ± 2.59 months, while for TB, it was 12.81 ± 6.17 months, with a significant difference between the groups (*p* < 0.05). The sex ratio in the CMA group consisted of 12 females and 10 males, while in the TB group, it was evenly split with 8 females and 8 males.

### 3.1. Comparison of Treatment Effects within the CMA Group (Table 2 and Table 3)

**Figure 2:**
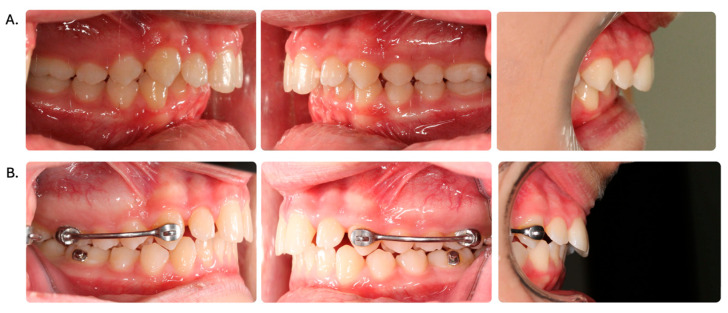
Class II correction with CMA before treatment (**A**) and after treatment (**B**).

The results indicate significant changes induced by the CMA, mainly in the dental variables (Figure 2). Although there were no significant changes in most skeletal variables, Wits appraisal demonstrated a significant decrease of 1.01 mm. The inclination of the upper incisors was not significantly affected. However, CMA treatment resulted in the significant protrusion of the lower incisors at 1.16 mm measured to the A-Pog line. The overjet and overbite were significantly reduced by 1.14 mm and 1.19 mm, respectively. The anteroposterior relation of the upper and lower molars improved significantly in both the distal aspect (DAMR −2.55 mm) and the mesial aspect (U6-L6—3.9 mm). Our results demonstrate a significant distalization (U6-NP) of 2.3 mm for the upper molar and a significant mesialization (L6-NP) of 1.6 mm for the lower molar, which includes a nonsignificant mandibular advancement (Pg—NP) of 0.71 mm. No significant changes in the soft tissue were observed. Table 3 presents the three variables contributing to the improvement in the molar relationship in the CMA group.

**Table 2:** Comparison of treatment effects within the CMA group.

Variable	Pre-Treatment		Post-Treatment		Difference	*p* Value
	Mean	SD	Mean	SD		
**Maxillary skeletal, sagittal**						
SNA ° ^2^	81.59	2.20	81.06	2.01	−0.53	0.18
A to Nasion Vertical, mm ^2^	0.12	2.34	−0.05	2.03	−0.16	0.71
Max. length, Co-A, mm ^2^	86.83	4.44	86.77	4.14	−0.06	0.93
**Mandibular skeletal, sagittal**						
SNB ° ^1^	76.84	2.76	76.77	2.65	−0.07	0.94
Pog to Nasion Vertical, mm ^2^	−7.57	5.61	−7.29	5.17	0.29	0.71
Mand. length Co-Gn, mm ^2^	107.54	6.22	108.50	6.41	0.96	0.41
**Maxillomandibular**						
ANB ° ^2^	4.76	1.68	4.29	1.92	−0.47	0.16
Wits mm ^1^	2.29	2.20	1.28	2.00	−1.01	0.042 *
Maxillomandibular differential mm ^1^	20.71	3.90	21.74	4.20	1.02	0.12
Angle of convexity ° ^2^	4.17	1.90	3.98	2.08	−0.19	0.55
**Vertical skeletal**						
SN-Mand. Pl. ° ^2^	36.24	4.48	36.43	5.01	0.19	0.67
Lower face height, mm ^2^	59.01	5.24	60.16	4.73	1.15	0.14
Face height ratio L\T % ^2^	52.71	2.08	53.04	2.04	0.33	0.25
**Dentoalveolar insicors**						
U1 to SN ° ^2^	102.41	6.43	102.01	6.73	−0.41	0.58
U1 to A-Pog, mm^2^	6.67	1.53	6.51	1.53	−0.16	0.41
L1 to Mand. Pl. ° ^2^	97.35	6.10	98.46	4.27	1.11	0.29
L1 to A-Pog, mm^2^	1.69	1.51	2.86	1.71	1.16	0.007 **
Interincisal angle ° ^2^	124.00	7.54	123.10	6.52	−0.90	0.53
Incisor overjet, mm ^1^	4.32	1.70	3.18	1.54	−1.14	0.0003 **
Incisor overbite, mm ^1^	3.80	2.09	2.61	2.19	−1.19	0.0054 **
**Dentoalveolar molars**						
DAMR, mm ^2^	0.17	2.24	−2.37	1.42	−2.55	5.2 × 10^−05^ **
U6-L6, mm^2^	0.06	1.13	−3.80	0.70	−3.9	4.8 × 10^−07^ **
NP-U6, mm^2^	27.54	3.62	29.90	3.76	2.3	4.8 × 10^−07^ **
NP-L6, mm^2^	27.60	3.83	26.00	3.84	−1.60	0.00029 **
A-NP, mm ^2^	0.00	2.34	−0.05	2.05	−0.05	0.913
Pg—NP, mm^2^	−7.95	5.47	−7.24	5.46	0.71	0.34
NP-UI, mm ^2^	−3.08	3.69	−3.39	3.45	−0.32	0.633
NP-LI, mm ^2^	1.31	3.24	−0.29	3.46	−1.60	0.018 **
Palatal Pl—U6 ° ^2^	83.40	4.93	77.42	4.93	−5.98	0.0002 **
SN—U6 ° ^2^	107.15	3.86	112.55	5.74	5.40	0.0002 **
S—U6, mm^2^	−61.99	4.61	−62.09	3.89	−0.10	0.88
S—L6, mm^2^	−61.78	4.57	−62.60	3.75	−0.81	0.24
**Soft tissue**						
Gl’-Sn’-Pog’ ° ^2^	17.69	5.42	16.66	4.86	−1.03	0.32
Nasolabial ° ^2^	99.34	9.41	101.84	10.92	2.50	0.28
Upper lip protrusion, mm ^2^	−2.97	2.82	−3.80	2.70	−0.83	0.19
Lower lip protrusion, mm ^2^	0.23	2.76	−0.74	3.42	−0.97	0.22
Upper 1 exposure, mm ^2^	5.67	2.14	5.56	3.18	−0.12	0.84
ST Na Perp-ST Pog, mm ^2^	−2.92	5.98	−2.48	5.67	0.44	0.63

Max indicates maxillar; Mand, mandibullar; U1, maxillary incisor; L1, mandibular incisor; U6, maxillary first molar; L6, mandibular first molar; and SD, standard deviation. Significance: * *p* < 0.05; ** *p* < 0.01. Wilcoxon signed-rank test ^1^ or paired t-test ^2^ was used to compare pre- and post-treatment

**Table 3:** The three variables contributing to the improvement the molar relationship in the CMA group.

		Measurement	Value
Measurements	Change in DAMR	U6-L6 distal side *	2.55 mm
	Change in molar relationship	U6-L6 mesial side *	3.9 mm
Variables	1. Distalization of the upper molar	U6 (mesial side) to NP	2.3 mm
	2. Mesialization of the lower molar	L6 (mesial side) to NP	1.6 mm
	3. Derotation	subtraction of U6-L6 to DAMR	1.35 mm

* See Table 1.

### 3.2. Comparison of Treatment Effects within the TB Group (Table 4)

The results within the TB group showed a statistically significant difference in the mandibular skeletal measurements, while no significant change was observed in the maxilla. Specifically, there was a significant increase of 1.05° in SNB and significant decreases of −1.69° and −2.62 mm in ANB and Wits appraisal, respectively. Furthermore, a significant increase of 3.49 mm in the skeletal maxillomandibular differential was found. In addition, a significant vertical change was observed as LFH increased (2.7 mm). Concerning the dentoalveolar effects of TB treatment, our findings indicate a statistically significant decrease in U1 to A-Pog (−162 mm), which suggests the retrusion of the upper incisors. Moreover, we observed a statistically significant increase in L1 to A-Pog of 2.62 mm, indicating the protrusion of the lower incisors. Furthermore, a significant reduction in both the overjet (−3.43 mm) and overbite (−3.04 mm) was found. The anteroposterior relationship of the upper and lower molars notably improved, with the correction of the molar relationship (DAMR) by 4.22 mm. Soft tissue measurements revealed a significant increase of 1.95 mm in the lower lip protrusion.

**Table 4:** Comparison of treatment effects within the TB group.

Variable	Pre-Treatment		Post-Treatment		Difference	*p* Value
	Mean	SD	Mean	SD		
**Maxillary skeletal, sagittal**						
SNA° ^2^	81.84	3.53	81.19	3.66	−0.65	0.23
A to Nasion Vertical, mm ^2^	2.34	3.93	1.21	4.28	−1.12	0.09
Max. length, Co-A, mm ^2^	90.11	6.87	89.60	6.46	−0.51	0.70
**Mandibular skeletal, sagittal**						
SNB ° ^2^	74.59	3.40	75.63	3.56	1.05	0.04 *
Pog to Nasion Vertical, mm ^2^	−8.34	5.36	−7.81	5.52	0.53	0.64
Mand. length Co-Gn, mm ^2^	107.92	8.21	110.91	7.24	2.98	0.09
**Maxillomandibular**						
ANB ° ^2^	7.25	1.66	5.56	1.69	−1.69	2.00 × 10^−04^ **
Wits mm ^2^	5.65	2.71	3.04	2.69	−2.62	2.00 × 10^−03^ **
Maxillomandibular differential mm ^2^	17.81	3.80	21.30	3.94	3.49	1.00 × 10^−03^ **
Angle of convexity ° ^2^	6.84	2.16	5.26	2.35	−1.58	3.00 × 10^−04^ **
**Vertical skeletal**						
SN-Mand. Pl. ° ^2^	36.45	5.56	36.63	5.78	0.18	0.75
Lower face height, mm ^2^	58.52	4.61	61.22	5.41	2.70	0.02 *
Face height ratio L\T % ^2^	52.76	2.56	53.60	3.05	0.84	0.04 *
**Dentoalveolar**						
U1 to SN ° ^2^	105.52	8.82	102.83	8.90	−2.69	0.10
U1 to A-Pog, mm ^1^	9.20	2.84	7.58	2.54	−1.62	4.00 × 10^−03^ **
L1 to Mand. Pl. ° ^2^	100.77	7.16	102.46	7.54	1.69	0.06
L1 to A-Pog, mm ^1^	0.79	2.57	3.41	2.13	2.62	9.16 × 10^−05^ **
Interincisal angle ° ^1^	117.27	11.32	118.08	11.43	0.82	0.75
Incisor overjet, mm ^1^	7.11	2.48	3.68	1.73	−3.43	2.10 × 10^−03^ **
Incisor overbite, mm ^2^	5.84	1.60	2.80	1.73	−3.04	5.00 × 10^−04^ **
DAMR, mm ^2^	0.57	2.62	−3.65	1.77	−4.22	3.00 × 10^−04^ **
**Soft tissue**						
Gl’-Sn’-Pog’ ° ^2^	20.05	3.98	19.97	5.05	−0.08	0.94
Nasolabial ° ^2^	99.32	12.05	102.60	13.96	3.28	0.23
Upper lip protrusion, mm ^2^	−1.32	2.90	−1.67	3.42	−0.35	0.64
Lower lip protrusion, mm ^2^	−0.74	2.59	1.21	3.14	1.95	4.40 × 10^−03^ **
Upper 1 exposure, mm ^2^	6.52	2.09	5.75	2.71	−0.76	0.31
ST Na Perp-ST Pog, mm ^2^	−4.23	5.30	−3.27	4.80	0.96	0.38

Max indicates maxillar; Mand, mandibullar; U1, maxillary incisor; L1, mandibular incisor; and SD, standard deviation. Significance: * *p* < 0.05; ** *p* < 0.01. ^1^ Wilcoxon signed-rank test or ^2^ paired *t*-test was used to compare pre- and post-treatment changes within the Twin Block group

### 3.3. Comparison between CMA and TB Groups (Table 5 and Table 6)

**Skeletal effect:** Both groups meet the definitions of skeletal Class II as reflected by the characteristics in Table 6. Post-treatment results indicated no significant difference between the groups in the position and size of the maxilla. In the mandible, a significant difference in SNB was shown between TB and CMA (1.05° and 0.07°, respectively). ANB and Wits appraisal demonstrated a decrease in both groups, with a significantly larger difference in the TB group. The CMA group showed a decrease of −0.47° in ANB and −1.01 mm in Wits, while the TB group exhibited a larger decline, with ANB decreasing by −1.69° and Wits by −2.62 mm. Within the CMA group, a nonsignificant increase of 1.02 mm in the maxillomandibular differential was found, while in the TB group, it increased significantly (3.49 mm). Additionally, a comparison between the two groups revealed significant differences. The angle of convexity was significantly decreased within the TB group (1.58°) and between both groups. No significant differences in the vertical skeletal variables were found (Table 5).

**Dental effect:** Both appliances retruded the upper incisor relative to A-Pog (CMA: 0.16 mm, TB: 1.62 mm) and significantly protruded the lower incisor (CMA: 1.16 mm, TB: 2.62 mm). A significant difference in lower incisors proclination (L1 to A-Pog) was observed between the groups. Both the overjet and the overbite decreased in the two groups, and this was observed significantly more in the TB group. Both appliances improved the molar relationship (DAMR) with a greater average improvement by the TB (4.22 mm compared to 2.55 mm in the CMA) (Table 5).

**Soft tissue effect:** The only change in soft tissue that was significantly different between the groups was observed in lower lip protrusion (CMA: −0.97 mm, TB: 1.95 mm) (Table 5).

**Table 5:** Comparison between CMA and TB groups (treatment effects).

Variable	Carrier N = 22		Twin Block N = 16		
	Mean	SD	Mean	SD	*p* Value
**Maxillary skeletal, sagittal**					
SNA ° ^2^	−0.54	1.82	−0.65	2.06	0.86
A to Nasion Vertical, mm ^2^	−0.16	2.08	−1.12	2.47	0.20
Max. length, Co-A, mm ^2^	−0.06	3.71	−0.51	5.09	0.76
Mandibular skeletal, sagittal					
SNB ° ^2^	−0.07	1.21	1.05	1.82	0.03 *
Pog to Nasion Vertical, mm ^2^	0.29	3.54	0.53	4.52	0.85
Mand. length Co-Gn, mm ^2^	0.96	5.34	2.98	6.76	0.31
**Maxillomandibular**					
ANB ° ^1^	−0.47	1.50	−1.69	1.05	0.01 *
Wits mm ^2^	−1.01	1.99	−2.62	2.89	0.05 *
Maxillomandibular differential mm ^2^	1.02	2.71	3.49	3.24	0.02 *
Angle of convexity ° ^2^	−0.19	1.49	−1.58	1.16	4.00 × 10^−03^ **
**Vertical skeletal**					
SN-Mand. Pl. ° ^1^	0.19	2.12	0.18	1.95	0.47
Lower face height, mm ^2^	1.15	3.53	2.70	3.95	0.21
Face height ratio L\T %. ^2^	0.33	1.32	0.84	1.50	0.27
**Dentoalveolar**					
U1 to SN ° ^2^	−0.41	3.23	−2.69	6.08	0.14
U1 to A-Pog, mm ^1^	−0.16	0.89	−1.62	1.80	2.00 × 10^−03^ **
L1 to Mand. Pl. ° ^1^	1.11	4.85	1.69	3.40	0.80
L1 to A-Pog, mm ^2^	1.16	1.80	2.62	1.56	1.20 × 10^−03^ **
Interincisal angle ° ^2^	−0.90	6.57	0.82	4.96	0.39
Incisor overjet, mm ^2^	−1.14	1.14	−3.43	2.29	3.00 × 10^−04^ **
Incisor overbite, mm ^2^	−1.19	1.76	−3.04	2.52	0.01 *
DAMR, mm ^2^	−2.55	2.29	−4.22	2.99	0.06
**Soft tissue**					
Gl’-Sn’-Pog’ ° ^1^	−1.03	4.73	−0.08	4.09	0.75
Nasolabial ° ^2^	2.50	10.69	3.28	10.49	0.82
Upper lip protrusion, mm ^1^	−0.83	2.85	−0.35	2.87	0.60
Lower lip protrusion, mm ^1^	−0.97	3.51	1.95	2.41	3.00 × 10^−03^ **
Upper 1 exposure, mm ^2^	−0.12	2.74	−0.76	2.82	0.48
ST Na Perp-ST Pog, mm ^2^	0.44	4.28	0.96	4.26	0.71

Max indicates maxillar; Mand, mandibullar; U1, axillary incisor; L1, mandibular incisor; and SD, standard deviation. Significance: * *p* < 0.05; ** *p* < 0.01. ^1^ Mann–Whitney U tests or ^2^ Student’s *t*-test was carried out to compare differences between the two groups.

**Table 6:** Comparison between CMA and TB groups (pre-treatment).

Variable	Carrier N = 22		Twin Block N = 16	
	Mean	SD	Mean	SD
**Maxillary skeletal, sagittal**				
SNA °	81.59	2.20	81.84	3.53
**Mandibular skeletal, sagittal**				
SNB °	76.84	2.76	74.59	3.20
**Maxillomandibular**				
ANB °	4.76	1.68	7.40	1.63
**Dentoalveolar**				
L1 to Mand. Pl. °	97.35	6.10	101.29	6.32
U1 to A-Pog, mm	6.67	1.53	9.42	2.82

Mand, mandibullar; L1, mandibular incisor; and SD, standard deviation. Student’s *t*-test was carried out to compare differences between the two groups.

## 4. Discussion

We evaluated the skeletal, dentoalveolar, and soft tissue changes induced by the CMA and the TB appliances. Both groups showed initial values that indicated dentoalveolar Class II. In addition, the data included skeletal Class II parameters (Table 6) as expressed by an ANB angle greater than 4° [20], an SNB angle less than 77°, and dentoalveolar compensation (U1 to A Pog above 6.5 mm, IMPA above 97°). Regarding the rising prevalence of the CMA in recent years [25], it was important to evaluate its mechanism of action. TB and CMA significantly improved DAMR (4.22 mm and 2.55 mm, respectively). Within the CMA group, the molar relationship (U6-L6) showed a total improvement of 3.9 mm (Table 3). This improvement comprised three variables. The first two were the upper molar distalization (measured by U6-NP of 2.3 mm) and the lower molar mesialization (measured by L6-NP of 1.6 mm), which included a skeletal component of mandibular advancement (Pg–NP:0.71 mm). The upper molar distalization involved a combination of anterior–posterior movements. It included linear and angular adjustments (U6-NP at 2.3 mm and SN-U6 angle at 5.4° (Table 2)), which correlates with findings in the literature [19,20]. However, a significant contribution to the correction of the molar relationship was attained by the third variable, which was the derotation of the upper molar. Although this derotation could not be directly quantified in a 2D cephalometric analysis, it can be estimated by the subtraction of the measurements regarding the mesial and the distal side of the maxillary first molar (see Table 3). The difference between the U6-L6 measurement of 3.9 mm (mesial side) and the DAMR parameter of 2.55 mm (distal side) can presumably be directly attributed to derotation. These three factors of the multidirectional correction of the molar relationship were highlighted in various studies [16,19,20,21]^.^ It is noteworthy to underline that the changes in the CMA group are comparable to those in other studies [22,26]. The disparities can be attributed to differences in measurement methodologies and study design.

The negligible skeletal effect on the maxilla induced by the CMA is comparable to the literature [16,19,21,22,26]. Additionally, similar changes that the TB and the CMA induced in the size of the maxilla (CoA: 0.51 mm, 0.06 mm, respectively) represent a minimal sagittal growth inhibition [22]. Our findings regarding the small mandibular skeletal changes caused by the CMA are either identical to other findings in the literature (SNB, Pog to Nasion Vertical) or even smaller (Co-Gn) [19,21,22,23]. Also, the nonsignificant reduction in ANB (−0.47°) and the significant reduction in Wits (−1.01 mm) in the CMA group are similar to findings in other studies (Wits: −0.5 to 2.1 mm, ANB:−0.5° to −0.8° [19,21,22,23,26]). TB showed an increase of 2.98 mm in mandibular length, which is about threefold more than the impact of the CMA. The maxillo-mandibular differential demonstrated a nonsignificant amount of change in the CMA group (1.02 mm) and was comparable to the results of Kim et al. [22] (1.7 mm). Upon reviewing the significant mandibular changes with the TB, it can be concluded that the CMA has a minimal functional impact on the mandible. The vertical changes in the TB and the CMA groups were both associated with molar extrusion, which can cause vertical skeletal changes. Since correction in molar relationships can be accompanied by their extrusion [27], which is not always compensated by growth, it is reasonable to expect a small vertical skeletal change. Indeed, the TB group induced a greater but statistically insignificant increase in LFH compared to the CMA (2.7 mm and 1.15 mm, respectively). This minor vertical change induced by the CMA was smaller than that reported in the literature [26]. Upper incisor retroclination was significantly greater in the TB compared to the CMA group (Upper 1 to A-Pog. −1.69 mm, −0.16 mm, respectively). This change in the TB group is explained by the headgear effect [10]. Care must be taken when assessing the change in the inclination of the upper incisors obtained in the CMA group, as no direct force is applied to them by this appliance. This finding could be advantageous for patients with a high risk of root resorption [21] and is consistent with the results reported in the literature [20,21,22]. In contrast, there are conflicting reports describing the proclination of the upper incisors by the CMA, which was accredited to a certain clinical situation of decreased overjet and lower incisor proclination, thereby causing the upper incisors to tip forward [20,23]. While both groups showed significant protrusion of the lower incisors, there was significantly less in the CMA group compared to the TB group (1.16 mm and 2.62 mm, respectively). Our findings of less loss of anchorage in the mandibular incisors caused by the CMA are consistent or even smaller than previous studies [15,17,19,20,22,23,26]. However, a significant anterior movement of the dentition and mandibular incisor protrusion is associated with other orthodontic modalities, such as the TB appliance [10], as well as fixed functional appliances [28], and Class II elastics [18]. It could potentially be found after Class II correction by the CMA. Nonetheless, the minor anchorage loss seen in the CMA group is presumably attributed to the relatively minimal retraction of the maxillary incisors, the reduced duration of elastic wear [26], and the use of an Essix retainer simultaneously with the CMA compared to the labial arch used with the TB. In this regard, a recent study introduced the use of infrazygomatic miniscrew-anchored CMA without any intervention in the mandible in order to prevent the side effects of Class II elastics regarding lower incisor proclination and the mesial movement of the lower molars [14]. When using the conventional CMA, special care should be taken when treating Class II patients with proclined lower incisors as it can influence the gingivae, especially in individuals with thin gingival biotypes [16]. The reduction in the overjet and overbite was significant for both appliances. These findings regarding the CMA are fairly similar to those reported in the literature [19,20,22,23,26]. In our study, the overjet correction attained using the CMA i exhibits a comparatively modest degree of adjustment relative to outcomes reported in other studies for alternative Class II corrective modalities, such as the Forsus [29] appliance and mini screws [13]. These latter approaches yield results that align more closely with the findings associated with the Twin Block appliance. Overjet and overbite reduction in the TB group can be attributed to both skeletal and dentoalveolar changes. In contrast, in the CMA group, these differences were primarily dentoalveolar since no major skeletal effects were found.

Our study revealed a notable increase in the protrusion of the lower lip among the TB group, and this change was also significant when comparing the CMA group to the TB group, as shown in Table 4 and Table 5. This change can be attributed to the skeletal and dental influences observed in the TB group, aligning with previous research findings in the literature [30].

In summary, the findings of this study confirm our null hypothesis that the primary treatment effect of the CMA is dentoalveolar. Furthermore, our analysis succeeded in representing the multidirectional upper and lower molar movements, highlighting the upper molar derotation contribution to the correction of Class II malocclusion by using 2D cephalometric analysis only. The limitations of the study include the following: (1) its retrospective design, (2) the unavailability of information about patient compliance, (3) the fact that the analysis was restricted to the initial treatment phase of using the CMA or the TB without encompassing observations during the subsequent treatment, and (4) 2D cephalometric radiographic images might involve the distortion and overlap of anatomical structures.

## 5. Conclusions

- The CMA is capable of correcting about 4 mm of the Class II dentoalveolar relationship, which is attributable to a linear and considerable rotational movement of maxillary molars.
- The skeletal effects and soft tissue alterations of the CMA were found to be less pronounced than the changes induced by the TB functional appliance.
- The CMA decreases the additional effects commonly associated with functional appliances, including an increase in LFH and lower incisor protrusion, which typically indicates loss of anchorage.
- OJ and OB are significantly reduced by both appliances but to a lesser extent by the CMA.
- Further research is needed to validate and substantiate the results of this study.

## Figures and Tables

**Table 1:** Definitions of the cephalometric measurements that were evaluated.

SNA°	The angle between the Sela, Nasion, and A point
A to Nasion Vertical, mm	Distance between point A and the Nasion Vertical line
Max. length, Co-A, mm	Distance between condilyon and point A
SNB °	The angle between the Sela, Nasion, and B point
Pog to Nasion Vertical, mm	Distance between point Pog (the most anterior point on the chin) and the Nasion Vertical line
Mand. length Co-Gn, mm	Distance between condilyon and point B
ANB °	The angle between Nasion, point A, and point B
Wits	BO-AO segment in which AO and BO indicate the projections of A and B points on the occlusal plane. Positive value when AO precedes BO
Maxillomandibular differential mm	The difference between the length of the mandible and the maxilla
Angle of convexity °	The angle formed between the N-A line and the line A-Pog
SN-Mand. Pl. °	Angle between the SN plane and the mandibular plane (Go—Gn)
Lower face height, mm	Distance between the Anterior Nasal Spine (ANS) and the Menton (Me)
Face height ratio L\T %	The ratio between lower face height (measured from Anterior Nasal Spine to Menton) and the total face height (measured from Nasion to Menton)
U1 to SN °	The angle between the long axis of the upper incisor (cusp tip to root apex) to the NS plane (anterior skull base)
U1 to A-Pog, mm	Distance between the most prominent point of the upper central incisor (Upper 1) and the A-Pog line, which connects point A (deepest midline point on the maxilla) to Pogonion (most anterior point on the bony chin)
L1 to Mand. Pl. °	IMPA. The angle between the mandibular plane and the axis of the inferior anterior incisor
L1 to A- Pog, mm	Distance between the most prominent point of the lower central incisor (Lower 1) and the A-Pog line, which connects point A (deepest midline point on the maxilla) to Pogonion (most anterior point on the bony chin)
Interincisal angle °	The angle formed by the intersection of the long axes of the upper central incisor (Upper 1) and the lower central incisor (Lower 1)
Incisor overjet, mm	Distance between incisal point of maxillary incisor and incisal point of mandibular incisor taken on a horizontal plane
Incisor overbite, mm	Distance between incisal point of maxillary incisor and incisal point of mandibular incisor taken on a vertical plane
DAMR, mm	Horizontal distance between the distal aspect of the maxillary first molar and the distal aspect of the mandibular first molar. Negative values mean either distalization of upper first molar or mesialization of first lower molar to achieve Class II correction
U6-L6, mm	Horizontal distance between the mesiobuccal cusp tips of the maxillary first molar (U6) and the mandibular first molar (L6)
NP-U6, mm	Horizontal distance between the nasion (NP) and the mesiobuccal cusp tip of the maxillary first molar (U6)
NP-L6, mm	Horizontal distance between the nasion (NP) and the mesiobuccal cusp tip of the mandibular first molar (L6)
Palatal Pl—U6 °	The angle formed by the intersection of the palatal plane (a line that passes through the anterior and posterior nasal spine and the middle of the tuberosity of the maxilla) and the long axis of the maxillary first molar (U6)
SN—U6 °	The angle measured through the long axis of the mesiobuccal cusp to the mesiobuccal root apex of the maxillary first molar in relation to the Sella–Nasion line viewed from the lateral (right and left)
S—U6, mm	Vertical distance between the Sella (S) and the mesiobuccal cusp tip of the maxillary first molar (U6)
S—L6, mm	Vertical distance between the Sella (S) and the mesiobuccal cusp tip of the mandibular first molar (L6)
Gl’-Sn’-Pog’ °	The angle formed between soft tissue Glabela, subnasale, and Pogonion
Nasolabial °	The angle formed between columella, subnasale, and Upper lip tip
Upper lip protrusion, mm	The linear distance between the E-line (a horizontal reference line passing through the tips of the upper and lower central incisors) and the most anterior point on the upper lip
Lower lip protrusion, mm	linear distance between the E-line and the most anterior point on the lower lip
Upper 1 exposure, mm	linear distance between the E-line and the incisal edge of the upper central incisor (Upper 1)
ST Na Perp-ST Pog, mm	Horizontal distance soft tissue nasion to the soft tissue Pogonion

## Data Availability

The data presented in this study are available on request from the corresponding author (to accurately indicate status).
